# Proteomic Profiling of Serum Biomarkers Associated with Dementia in an Egyptian Cohort

**DOI:** 10.1002/alz70856_107468

**Published:** 2026-01-09

**Authors:** Shimaa Adel Heikal, Mohamed Salama

**Affiliations:** ^1^ The American University in Cairo, Cairo, Egypt; ^2^ Institute of Global Health and Human Ecology at the American University in Cairo, Cairo, Cairo, Egypt

## Abstract

**Background:**

Dementia and aging problems are currently growing worldwide and are considered public health priorities. It is a complex neurodegenerative disorder influenced by multiple pathophysiological processes. Identifying specific biomarkers for the early detection of Dementia is crucial, particularly in underrepresented populations such as the Egyptian cohort.

**Method:**

As part of the development of a national dementia registry for Egypt that would be of great importance, the present study aims to perform proteomic profiling to identify serum biomarkers associated with Dementia in an Egyptian cohort, providing insights into disease mechanisms and potential diagnostic markers. We analyzed 30 serum samples including patients and controls, recruited by the Egyptian Dementia Registry (EDN). We assessed the differences in protein expression between patients and controls to identify the dysregulated biological pathways.

**Result:**

Our findings reported 222 unique proteins, 41 were significantly differentially expressed between Dementia patients and controls (*p* < 0.05). Some proteins were upregulated in patients such as Complement factor I and Antithrombin‐III and others were downregulated including Plasma protease C1 inhibitor and Lipopolysaccharide‐binding protein. Enrichment analysis linked these proteins to pathways implicated in inflammatory response, complement activation, coagulation, and fibrin clot formation.

**Conclusion:**

In conclusion, this study provides the first proteomic characterization of an Egyptian Dementia cohort, identifying novel and established biomarkers associated with the disease. The results underscore the importance of inflammatory and immune pathways in disease pathogenesis and highlight population‐specific proteomic signatures.

